# The HARBINGER of endoscopic therapy in critically-ill patients with upper GI bleeding

**DOI:** 10.1093/gastro/goaa071

**Published:** 2020-12-03

**Authors:** Bazerbachi Fateh, Horibe Masayasu

**Affiliations:** 1 Division of Gastroenterology, Department of Medicine, Massachusetts General Hospital, 55 Fruit Street, Boston, MA, USA; 2 Department of Internal Medicine, Division of Gastroenterology and Hepatology, Keio University School of Medicine, Shinjuku-ku, Tokyo, Japan; 3 Division of Gastroenterology and Hepatology, Mayo Clinic, 200 First St SW, Rochester, MN, USA

We read with great interest the study by Rao et al. [1] Rao and colleagues showed that the Rockall score, Glasgow-Blatchford score (GBS) and AIMS65 are poorly predictive of 30-day mortality, or the need for endoscopic intervention in intensive care unit (ICU) patients with an upper GI bleed (UGIB).

The Rockall [2] score and AIMS65 [3] were developed for predicting mortality, whereas the GBS [4] was developed for predicting composite outcomes (The need for a blood transfusion or intervention to control bleeding, rebleeding, or death)

We were not surprised to see that these scores were poor predictors of endoscopic intervention, as they were not designed to predict high-risk endoscopic stigmata (HRS), which is arguably a very meaningful endpoint to determine the need for endoscopic intervention.

Recently, a simple score (Horibe gastrointestinal bleeding prediction score [HARBINGER]) was developed to predict the presence HRS [5], and was prospectively validated in Japanese patients [6]. The HARBINGER consists of only three variables (score, 0–3 points): i) absence of daily proton pump inhibitors (PPI) use in the week before the index presentation (1 point); ii) shock index (heart rate [HR]/systolic blood pressure [SBP]) ≥ 1 (1 point); and iii) blood urea nitrogen/creatinine ≥ 30 [urea/ creatinine ≥140] (1 point). The HARBINGER provide a more accurate method for triage of patients with suspected UGIB than both the GBS and the AIMS65, and an urgent endoscopy is sought for those with a HARBINGER ≥ 2.

In the HARBINGER validation study, the evaluation of HRS was done by expert endoscopists, blinded to the clinical information of patients to reduce the risk of bias [6]. Although we did not perform a subgroup analysis of ICU patients, we have shown that HARBINGER can significantly predict HRS better than both scores even patients deemed to be of very high risk ( those with a GBS ≥ 12 or an AIMS65 ≥ 2). The HARBINGER may thus play a role in the triage of ICU patients [6].

Rao et al. [1] highlighted the need for a more accurate risk-stratification tool to predict the benefit of intervention within the ICU population, and we believe that the HARBINGER might meet their needs. If Rao and colleagus [1] possess data regarding PPI use, as well as provide a BUN/Cr ratio, they would be able to perform an exploratory analysis to assess the performance of the HARBINGER with HRS as the outcome, rather than any delivery of endoscopic treatment (which can be an operator-depedent variable).

Of note, we completely agree with the proposed differentiation between patients admitted to the ICU with UGIB and those who have been already in the ICU and later are suspected of having developed UGIB (e.g. coffee-ground suction from an orogastric tube); capturing the time of hospital admission, ICU admission, and time of endoscopy is key to separate these phenotypes.

Rao et al. [1] are to be commended for addressing a very important clinical issue, and we would be eager to see an exploratory analysis application of the HARBINGER to their cohort of critically ill patients. However, we understand that, given the limitations of granular data, perfect retrospective application of the HARBINGER may not be feasible.
